# Discovery and Characterization of Proteins Associated with Aflatoxin-Resistance: Evaluating Their Potential as Breeding Markers

**DOI:** 10.3390/toxins2040919

**Published:** 2010-04-26

**Authors:** Robert L. Brown, Zhi-Yuan Chen, Marilyn Warburton, Meng Luo, Abebe Menkir, Ahmad Fakhoury, Deepak Bhatnagar

**Affiliations:** 1 USDA-ARS, Southern Regional Research Center, New Orleans, LA 70124, USA; Email: deepak.bhatnagar@ars.usda.gov; 2 Department of Plant Pathology and Crop Physiology, Louisiana State University Agricultural Center, Baton Rouge, LA 70803, USA; Email: zchen@agcenter.lsu.edu (Z.-Y.C.); meng.luo@ars.usda.gov (M.L.); 3 USDA-ARS, Mississippi State University, MS, USA; Email: marilyn.warburton@ars.usda.gov; 4 International Institute of Tropical Agriculture, Ibadan, Nigeria; Email: a.menkir@cgiar.org; 5 Department of Plant, Soil, and Agricultural Systems, Southern Illinois University, Carbondale, IL 62901, USA; Email: amfakhou@siu.edu

**Keywords:** aflatoxin-resistance, maize resistance, proteomics, breeding markers

## Abstract

Host resistance has become a viable approach to eliminating aflatoxin contamination of maize since the discovery of several maize lines with natural resistance. However, to derive commercial benefit from this resistance and develop lines that can aid growers, markers need to be identified to facilitate the transfer of resistance into commercially useful genetic backgrounds without transfer of unwanted traits. To accomplish this, research efforts have focused on the identification of kernel resistance-associated proteins (RAPs) including the employment of comparative proteomics to investigate closely-related maize lines that vary in aflatoxin accumulation. RAPs have been identified and several further characterized through physiological and biochemical investigations to determine their causal role in resistance and, therefore, their suitability as breeding markers. Three RAPs, a 14 kDa trypsin inhibitor, pathogenesis-related protein 10 and glyoxalase I are being investigated using RNAi gene silencing and plant transformation. Several resistant lines have been subjected to QTL mapping to identify loci associated with the aflatoxin-resistance phenotype. Results of proteome and characterization studies are discussed.

## 1. Introduction

Aflatoxins, the toxic and highly carcinogenic secondary metabolites of *Aspergillus flavus*, *A. parasiticus* and a number of fungi in the genus *Aspergillus*, are the most widely investigated of all mycotoxins due to their role in establishing the significance of mycotoxins in animal diseases, and to the regulation of their presence in food [1,2]. Aflatoxins pose serious health hazards to humans and domestic animals, because they frequently contaminate agricultural commodities [3,4]. Presently, a significant number of countries have established or proposed regulations for controlling aflatoxins in food and feeds [5]; the US Food and Drug Administration (FDA) has limits of 20 ppb, total aflatoxins, on interstate commerce of food and feed, and 0.5 ppb of aflatoxin M_1_ on the sale of milk. However, many countries, especially in the developing world, experience contamination of domestic-grown commodities to alarmingly greater levels than does the US A study revealed a strong association between exposure to aflatoxin and both stunting (a reflection of chronic malnutrition) and being underweight (a reflection of acute malnutrition) in West African children [6]. A 2004 outbreak of acute aflatoxicosis in Kenya, due to ingestion of contaminated maize, resulted in 125 deaths [7]. 

Recognition of the need to control aflatoxin contamination of food and feed grains has elicited various approaches from researchers to eliminate this toxin from maize and other susceptible crops. The approach to enhance host resistance through conventional or molecular breeding has gained renewed attention following the discovery of natural resistance to *A. flavus* infection and aflatoxin production in maize [8,9,10,11,12,13,14]. 

During the past two decades, maize genotypes with natural preharvest resistance to aflatoxin production have been identified through field screening [11,12,15]. The poor agronomic quality of these lines, however, renders them of little direct commercial value [14]. The lack of identified markers has slowed the incorporation of resistance into lines with commercially-acceptable genetic backgrounds. 

The expression of maize kernel proteins has been implicated in kernel resistance to *A. flavus* infection/aflatoxin production [16,17,18,19]. Using reverse genetics to identify genes that are associated with aflatoxin-resistance may lead to the discovery of breeding markers. These protein/gene markers could be used to transfer resistance to good genetic backgrounds while excluding undesirable traits. The purpose of this review is to highlight the discovery of resistance-associated proteins (RAPs) and their characterization as potential breeding markers.

## 2. Early Identification of Resistance-Associated Proteins (RAPs)

The development of a laboratory kernel screening assay (KSA) by Brown *et al*. [13] facilitated the verification of maize kernel resistance under laboratory conditions in a short time. This accelerated the discovery of knowledge surrounding host resistance mechanisms. Using this assay, Brown *et al*. [20] discovered the existence of subpericarp resistance in maize kernels and that the expression of this resistance requires a live embryo, indicating a potential role for kernel proteins in resistance. Guo *et al*. [18] found that imbibition of kernels, before inoculation with *A. flavus*, significantly increased aflatoxin-resistance of susceptible maize genotypes. Further investigation revealed that susceptible genotypes were able to induce antifungal proteins upon fungal infection [18], suggesting that susceptible lines have the ability to induce an active defense mechanism after fungal infection. The usefulness of the KSA as an investigative tool is aided by the fact that KSA results correlate well with field results [13] and that aflatoxin buildup occurs after kernel maturity, a developmental phase where constitutive factors required for kernel resistance are highlighted by the KSA [21]. However, resistance must be confirmed in the field; here, agronomic factors contributing to resistance can also be investigated. 

Examination of kernel proteins of several maize genotypes revealed differences between genotypes resistant or susceptible to aflatoxin contamination [22,23]. Imbibed susceptible kernels showed decreased aflatoxin levels and contained germination-induced ribosome inactivating protein (RIP) and zeamatin; both proteins have demonstrated growth-inhibitory activity *in vitro* against *A. flavus* [22]. In another study, two kernel proteins were identified from a resistant corn inbred line (Tex6), which may contribute to resistance to aflatoxin contamination [19]. When a commercial maize hybrid was inoculated with toxgenic and atoxigenic strains of *A. flavus* at milk stage, one chitinase and one β-1,3-glucanase isoform were detected in maturing infected kernels, while another isoform was detected in maturing uninfected kernels [24]. Lazovoya [25] reported that the presence of *A. flavus* caused an increase in β-1,3-glucanase activity in callus tissues from a resistant genotype, but not from a susceptible one. A more rapid and stronger induction of the PR-1 and PR-5 genes in maize leaves has also been observed in an incompatible interaction when compared to a compatible interaction upon pathogen infection [26]. A 14 kDa trypsin inhibitor protein (TI) was found to express at high levels in resistant lines but at low levels or is missing in susceptible ones [27]. This protein demonstrated antifungal activity against *A. flavus* and several other pathogenic fungi [28], possibly through inhibition of fungal α-amylase activity and production [29]. This could limit the availability of simple sugars needed for fungal growth and aflatoxin production [30]. 

The above-studies indicate an important role for kernel proteins in disease resistance. Further investigation, supporting earlier work by Guo [18], found that both constitutive and inducible proteins are required for kernel resistance to *A. flavus* infection and aflatoxin production [21]. This work showed that one major difference between resistant and susceptible genotypes is that resistant lines constitutively express higher levels of antifungal proteins compared to susceptible lines. Therefore, research on resistance genes/proteins has focused heavily on the identification of constitutively-produced kernel resistance-associated proteins (RAPs). 

## 3. Identification of RAPs through Comparative Proteomics

To increase protein resolution and detection sensitivity by 10 to 20 fold and, thus, enhance ability to identify more constitutively-expressed RAPs, proteomics approaches have been employed. Kernel proteins from several resistant and susceptible genotypes were compared using large format 2-D gel electrophoresis. A number of protein spots, either unique or 5-fold up-regulated in resistant lines, were detected, isolated from preparative 2-D gels and identified using ESI-MS/MS after in-gel digestion with trypsin [31,32]. These proteins can be grouped into three categories based on their peptide sequence homology: (1) storage proteins, such as globulins (GLB1, GLB2), and late embryogenesis abundant proteins (LEA3, LEA14); (2) stress-responsive proteins, such as aldose reductase (ALD), glyoxalase I (GLX I) and heat shock proteins, and (3) antifungal proteins, including TI. In total, approximately 21 proteins upregulated in resistant *versus* susceptible lines have been identified using comparative proteomics (Table 1). 

**Table 1:** RAPs identified through comparative proteomics ^1^.

RAPs	CITATION #
**Antifungals**	
Zeamatin	[21]
Trypsin inhibitor 14kDa (TI)	[26,32]
Ribosome inactivating protein (RIP)	[21,22]
β-1,3-glucanase	[38]
Pathogenesis-related protein 10 (PR10)	[44]
PR10.1	[45]
Trypsin inhibitor 10 kDa (ZmTI)	[55]
**Stress-related**	
Aldose Reductase (ALD)	[31]
Cold-regulated protein (ZmCORp)	[54]
Water stress inducible protein (WSI)	[31]
Anionic peroxidase	[32]
Small heat shock protein 16.9/17.2 kDa	[31,32]
Glyoxalase I (GLX I)	[43]
Peroxiredoxin 1 (PER1)	[32]
**Storage**	
Globulin I	[31]
Globulin II	[31,32]
Cupin domain containing protein (Zmcup)	[38]
Late embryogenesis abundant protein (LEA III)	[31,32]
LEA 14	[31,32]
**Other**	
Serine/threonine protein kinase	[38]
Translation initiation factor 5A	[38]

**^1 ^** Table is adaptation and updated version of table from reference #33.

No investigation has been conducted to determine the possible direct involvement of stress-related proteins in host fungal resistance. However, increased temperatures and drought, which often occur together, are major factors associated with aflatoxin contamination of corn kernels [34]. Unique or higher levels of hydrophilic storage or stress-related proteins, such as the aforementioned, may put resistant lines at an advantage for the ability to synthesize proteins and defend against pathogens while under stress. Further studies including physiological and biochemical characterization, genetic mapping, plant transformation using RAP genes, RNAi gene silencing experiments [35] and marker-assisted breeding should clarify the roles of stress-related RAPs in kernel resistance. 

To conduct the above-described comparative proteome studies, composite profiles for resistance and for susceptibility were developed from 2 D gels of several resistant or susceptible maize lines. This was done to homogenize nonresistance-related differences among lines within each group, and, therefore, facilitate the identification of resistance-related proteins. In using the composite gel approach, only those proteins that were five-fold upregulated in resistant *versus* susceptible lines were studied to minimize the chance of identifying proteins unrelated to host resistance. 

### 3.1. Closely-related lines

Recently, the screening of progeny generated through a collaborative breeding program between IITA-Nigeria (International Institute of Tropical Agriculture) and the Southern Regional Research Center of USDA-ARS in New Orleans (SRRC) facilitated the identification of closely-related lines from the same backcross differing significantly in aflatoxin accumulation, and proteome analysis of these lines is being conducted [36,37]. Investigating corn lines sharing close genetic backgrounds should enhance the identification of RAPs without the confounding effects experienced with lines of diverse genetic backgrounds. 

The IITA-SRRC collaboration has attempted to combine resistance traits of the U.S. resistant inbred lines with those of African lines, originally selected for resistance to ear rot diseases and for demonstrated potential aflatoxin-resistance (*via* KSA) [36,37]. Five elite tropical inbred lines from IITA adapted to the Savanna and mid-altitude ecological zones of West and Central Africa were crossed with four US resistant maize lines in Ibadan, Nigeria. The five African lines were originally selected for their resistance to ear rot caused by *Aspergillus*, *Botrydiplodia*, *Diplodia*, *Fusarium*, and *Macropomina* [36,37]. The F1 crosses were backcrossed to their respective US inbred lines and self-pollinated thereafter. The resulting lines were selected through the S4 generation for resistance to foliar diseases and desirable agronomic characteristics under conditions of severe natural infection in their respective areas of adaptation. Promising S5 lines were screened with the KSA. Five pairs of closely-related lines were shown to be significantly different in aflatoxin resistance, while sharing as high as 97% genetic similarity [38]. Using these lines in proteomic comparisons to identify RAPs has advantages: (1) gel comparisons and analyses become easier; and (2) protein differences between resistant and susceptible lines as low as twofold can be identified with confidence. In addition, the likelihood of identifying proteins that are directly involved in host resistance is increased. 

In a preliminary proteomics comparison of constitutive protein differences between those African closely-related lines, a new category of resistance-associated proteins (putative regulatory proteins) was identified, including a serine/threonine protein kinase and a translation initiation factor 5A [38]. The genes encoding these two resistance associated regulatory proteins are being cloned and their potential role in host resistance to *A. flavus* infection and aflatoxin production will be further investigated. 

Conducting proteomic analyses using lines from this program not only enhances chances of identifying genes important to resistance, but may have immediate practical value. The IITA-SRRC collaboration has recently registered and released six inbred lines with aflatoxin-resistance in good agronomic backgrounds, which also demonstrate good levels of resistance to southern corn blight and southern corn rust [39]. Resistance field trials for these lines on US soil will be conducted; the ability to use resistance in these lines commercially will depend on having identified excellent markers, since seed companies desire insurance against the transfer of undesirable traits into their elite genetic backgrounds. The fact that this resistance is coming from good genetic backgrounds is also a safeguard against the transfer of undesirable traits.

### 3.2. Proteome analysis of maize rachis and silk tissues

A study was conducted to investigate the proteome of rachis tissue, maternal tissue that supplies nutrients to the kernels [40]. An interesting finding in this study is that after infection by *A. flavus*, rachis tissue of aflatoxin-resistant genotypes did not up-regulate PR proteins as these were already high in controls where they had strongly and constitutively accumulated during maturation. However, rachis tissue of aflatoxin-susceptible lines did not accumulate PR proteins to such an extent during maturation, but increased them in response to fungal infection. Given the relationship of the rachis to kernels, these results confirm findings of Chen *et al*. [22], who demonstrated that higher constitutive levels of proteins in resistant *versus* susceptible kernels was a primary factor that determined kernel genetic resistance to aflatoxin contamination. 

Another study was conducted to identify proteins in maize silks that may be contributing to resistance against *A. flavus* infection/colonization [41]. Antifungal bioassays were performed using silk extracts from two aflatoxin-resistant and two–susceptible inbred lines. Silk extracts from resistant inbreds showed greater anti-fungal activity compared to susceptible inbreds. Comparative proteomic analysis of the two resistant and susceptible inbreds led to the identification of antifungal proteins including three chitinases that were differentially-expressed in resistant lines. When tested for chitinase activity, silk proteins from extracts of resistant lines also showed significantly higher chitinase activity than that from susceptible lines. Differential expression of chitinases in maize resistant and susceptible inbred silks suggests that these proteins may contribute to resistance.

## 4. Further Characterization of RAPs

A literature review of the RAPs identified above indicates that storage and stress-related proteins may play important roles in enhancing stress tolerance of host plants. The expression of storage protein GLB1 and LEA3 has been reported to be stress-responsive and ABA-dependant [42]. Transgenic rice overexpressing a barley LEA3 protein HVA1 showed significantly increased tolerance to water deficit and salinity [43]. 

The role of GLX I (Table 2) in stress-tolerance was first highlighted in an earlier study using transgenic tobacco plants overexpressing a *Brassica juncea* glyoxalase I [44]. The substrate for glyoxalase I, methylglyoxal, is a potent cytotoxic compound produced spontaneously in all organisms under physiological conditions from glycolysis and photosynthesis intermediates, glyceraldehydes-3-phosphate and dihydroxyacetone phosphate. Methylglyoxal is an aflatoxin inducer even at low concentrations; experimental evidence indicates that induction is through upregulation of aflatoxin biosynthetic pathway transcripts including the *AFLR* regulatory gene [45]. Therefore, glyoxalase I may be directly affecting resistance by removing its aflatoxin-inducing substrate, methylglyoxal. 

**Table 2:** Characterization of RAPs for breeding markers ^1^.

Protein	Associated w/resistance ^2^	Inhibitory of *A. flavus*	Enzymatic Activities	Expressed in development	RNAi silencing	Interact w/protein
TI-14 kD	UpReg. in **R**	+-high	Inhib. amylase; trypsin	Nda	Suscept	nda
PR-10	“	+ ^3^	RNase	10-fold upreg	Suscept	“
PR10.1	“	+	”	Nda	nda	“
ZmCORp	“	+	Lectin	“	“	“
ZmTI (10)	“	+-low	Inhib. trypsin	“	“	“
Zeamatin	“	+-low	Inhib. trypsin	“	“	“
β-1,3-Glu	“	+	Glucanase	“	“	“
GLX I	“	nda	Forms D-lactate	+	+	“
PER 1	“	“	Peroxidase	A.f. induced	nda	“
ALD	“	“	Reductase	Nda	“	“
RIP	“	+	Lytic	“	“	“
ZmCup	“	nda	nda	“	“	+

1. Investigative criteria for determining RAP involvement in resistance include upregulation in resistant *vs*. susceptible line, antifungal activity *vs*. *A. flavus*, enzymatic activities, expression level in kernels during development, kernel response to RNAi silencing, and interactions with other proteins. 2. All RAPs from Table 1, but not listed here, are also upregulated in resistant maize lines (**R**) *vs.* susceptible. 3. + denotes presence of activity; nda denotes no data available.

PER1, a 1-cys peroxiredoxin antioxidant identified in a proteomics investigation [32], was demonstrated to be an abundant peroxidase, and may play a role in the removal of reactive oxygen species. The PER1 protein overexpressed in *Escherichia coli* demonstrated peroxidase activity *in vitro*. It is possibly involved in removing reactive oxygen species produced when maize is under stress conditions [32].

Another RAP that has been characterized further is the pathogenesis-related protein 10 (PR10) (Table 2). It showed high homology to PR10 from rice (85.6% identical) and sorghum (81.4% identical). It also shares 51.9% identity to intracellular pathogenesis-related proteins from lily (AAF21625) and asparagus (CAA10720), and low homology to a RNase from ginseng [46]. The PR10 overexpressed in *E. coli* exhibited ribonucleolytic and antifungal activities. In addition, an increase in the antifungal activity against *A. flavus* growth was observed in the leaf extracts of transgenic tobacco plants expressing maize *PR10* gene compared to the control leaf extract [46]. This evidence suggests that PR10 plays a role in kernel resistance by inhibiting fungal growth of *A. flavus*. Further, its expression during kernel development was induced in the resistant line GT-MAS:gk, but not in susceptible Mo17 in response to fungal inoculation [46]. Recently, a new *PR10* homologue was identified from maize (*PR10.1*) [47]. *PR10* was expressed at higher levels in all tissues compared to *PR10.1*, however, purified PR10.1 overexpressed in *E. coli* possessed 8-fold higher specific RNase activity than *PR10* [47]. This homologue may also play a role in resistance.

Evidence supporting a role for *PR10* in host resistance is also accumulating in other plants. A barley *PR10* gene was found to be specifically induced in resistant cultivars upon infection by *Rhynchosporium secalis*, but not in near-isogenic susceptible plants [48]. In cowpea, a *PR10* homolog was specifically up-regulated in resistant epidermal cells inoculated with the rust fungus *Uromyces vignae* Barclay [49]. A *PR10* transcript was also induced in rice during infection by *Magnaporthe grisea* [50].

To directly demonstrate whether selected RAPs play a key role in host resistance against *A. flavus* infection, an RNA interference (RNAi) vector to silence the expression of endogenous RAP genes (such as *PR10*, *GLX I* and *TI*) in maize through genetic engineering was constructed [51,52]. The degree of silencing using RNAi constructs is greater than that obtained using either co-suppression or antisense constructs, especially when an intron is included [53]. Interference of double-stranded RNA with expression of specific genes has been widely described [54,55]. Although the mechanism is still not well understood, RNAi provides an extremely powerful tool to study functions of unknown genes in many organisms. This posttranscriptional gene silencing (PTGS) is a sequence-specific RNA degradation process triggered by a dsRNA, which propagates systemically throughout the plant, leading to the degradation of homologous RNA encoded by endogenous genes, and transgenes.

Both particle bombardment and *Agrobacterium*-mediated transformation methods were used to introduce the RNAi vectors into immature maize embryos. The former was used to provide a quick assessment of the efficacy of the RNAi vector in gene silencing. The latter, which can produce transgenic materials with fewer copies of foreign genes and is easier to regenerate, was chosen for generating transgenic kernels for evaluation of changes in aflatoxin-resistance. It was demonstrated using callus clones from particle bombardment that *PR10* expression was reduced by an average of over 90% after the introduction of the RNAi vector [52]. The transgenic kernels also showed a significant increase in susceptibility to *A. flavus* infection and aflatoxin production. The data from this RNAi study clearly demonstrated a direct role for PR10 in maize host resistance to *A. flavus* infection and aflatoxin contamination [52].

RNAi vectors to silence other RAP genes, such as *GLX I* and *TI*, have also been constructed, and introduced into immature maize embryos through both bombardment and *Agrobacterium* infection [56]. It will be very interesting to see the effect of silencing the expression of these genes in the transgenic kernels on host resistance to *A. flavus* infection and aflatoxin production.

ZmCORp, a protein with a sequence similar to cold-regulated protein and identified in the above-proteomic studies, was shown to exhibit lectin-like hemagglutination activity against fungal conidia and sheep erythrocytes [57]. When tested against *A. flavus*, ZmCORp inhibited germination of conidia by 80% and decreased mycelial growth by 50%, when germinated conidia were incubated with the protein. Quantitative real-time RT-PCR revealed *ZmCORp* to be expressed 50% more in kernels of a resistant maize line *versus* a susceptible.

ZmTIp, a 10 kDa trypsin inhibitor, had an impact on *A. flavus* growth, but not as great as the previously-mentioned 14 kDa TI [58]. 

### 4.1. Gene mapping

Chromosome regions associated with resistance to *A. flavus* and inhibition of aflatoxin production in maize have been identified through Restriction Fragment Length Polymorphism (RFLP) analysis in three “resistant” lines (R001, LB31, and Tex6) in an Illinois breeding program, after mapping populations were developed using B73 and/or Mo17 elite inbreds as the “susceptible” parents [59,60]. Chromosome regions associated with inhibition of aflatoxin in studies considering all 3 resistant lines demonstrated that there are some chromosome regions in common. Regions on chromosome arms 2L, 3L, 4S, and 8S may prove promising for improving resistance through marker assisted breeding into commercial lines [60]. In some cases, chromosomal regions were associated with resistance to *Aspergillus* ear rot and not aflatoxin inhibition, and vice versa, whereas other chromosomal regions were found to be associated with both traits. This suggests that these two traits may be at least partially under separate genetic control. 

QTL studies involving other populations have identified chromosome regions associated with low aflatoxin accumulation. In a study involving 2 populations from Tex6 x B73, conducted in 1996 and 1997, promising QTLs for low aflatoxin were detected in bins 3.05-6, 4.07-8, 5.01-2, 5.05-5, and 10.05-10.07 [61]. Environment strongly influenced detection of QTLs for lower toxin in different years; QTLs for lower aflatoxin were attributed to both parental sources. In a study involving a cross between B73 and resistant inbred Oh516, QTL associated with reduced aflatoxin were identified on chromosomes 2, 3 and 7 (bins 2.01 to 2.03, 2.08, 3.08, and 7.06) [62]. QTLs contributing resistance to aflatoxin accumulation were also identified using a population created by B73 and resistant inbred Mp313E, on chromosome 4 of Mp313E [63]. This confirmed the findings of an earlier study involving Mp313E and susceptible Va35 [64]. Another QTL in this study, which has similar effects to that on chromosome 4, was identified on chromosome 2 [63]. A recent study to identify aflatoxin-resistance QTL and linked markers for marker-assisted breeding was conducted using a population developed from Mp717, an aflatoxin-resistant maize inbred, and NC300, a susceptible inbred adapted to the southern US QTL were identified on all chromosomes, except 4, 6, and 9; individual QTL accounted for up to 11% of phenotypic variance in aflatoxin accumulation [65]. 

A number of RAP genes identified in the proteomics studies have been mapped to chromosomal location (Table 3) using the genetic sequence of B73 now available online (http://archive.maizesequence.org/index.html). Using the DNA sequence of the RAPs and blasting them against the B73 sequence allowed us to place each gene into a virtual Bin, allowing us to pinpoint the chromosomal location to which each gene maps. The chromosomes involved include the above-mentioned chromosomes 1, 2, 3, 7, 8 and 10, some in bins closely located to those described above. This adds support to proteomic data and characterization results that suggest the involvement of 14 kDa TI, water stress inducible protein, zeamatin, one of the heat shock, cold-regulated, glyoxalase I and PR10 proteins in aflatoxin-resistance. From the above QTL investigations, it is observed that variation can exist in the chromosomal regions associated with *Aspergillus* ear rot and aflatoxin inhibition in different mapping populations. This suggests the presence of different genes for resistance in the different identified resistant germplasm. It will be important to map resistant lines investigated through proteomics or to obtain data from associative mapping panels regarding gene location.

**Table 3:** Mapping of RAPs on maize chromosomes ^1^.

RAP Gene	NCBI Accession Number	Bin
Zeamatin	AAB21820.1	7.04
TI-14 kDa	X54064	2.06
PR10	AY953127	1.03
GLX I	AY241545	10.3
ZmCOR	CK986091	8.04
WSI	BAA05537	3.07
Heat shock	AW258080	1.03
Heat shock	BE123268	8.01

^1 ^Mapping information obtained through Maize Genome Browser, Maize Sequence version 3b.50, http://archive.maizesequence.org/index.html.

## 5. Conclusions

Host resistance as a strategy for eliminating aflatoxin contamination of maize is closer to being a reality due to the identification of genotypes with natural resistance to aflatoxin accumulation and the development of new inbred lines through breeding. However, to exploit this resistance for the benefit of maize growers, markers have to be identified to facilitate the transfer of resistance to elite proprietary backgrounds currently in commercial use. The identification of resistance-associated proteins goes a long way towards providing the novel markers that will be indispensible to any commercial breeding undertaking. Characterization studies including RNAi gene silencing and gene mapping are instrumental in building a case for the involvement of selected RAPs in kernel resistance to aflatoxin contamination. 

Several publications by the current authors have profiled many of the RAPs listed in the present review. Here, however, the most complete listing of RAPs identified through comparative proteomics is presented along with available evidence of their potential as breeding markers. Investigations of RAPs, as discussed above, not only impact the development of commercially-useful resistant maize lines, but provide an expanding base of knowledge concerning nature’s requirements for creating a durable resistance against the opportunistic pathogen, *A. flavus*. It remains to be determined, how the different categories of proteins, antifungal, stress-related, storage and others contribute to the total picture of resistance. Future investigations (e.g., proteomics and microarray analysis) may also impact aflatoxin-resistance through the discovery of RAPs down-regulated in resistant lines, RAPs induced upon fungal infection and also factors involved in the regulation of RAPs. These discoveries will not only contribute to the development of aflatoxin-resistant maize lines, they may aid other susceptible crops, assist in meeting the challenges of other mycotoxin-producing fungi, while enhancing our understanding of host plant interactions with fungi. 
